# Low dose Intralipid resuscitation improves survival compared to ClinOleic in propranolol overdose in rats

**DOI:** 10.1371/journal.pone.0202871

**Published:** 2018-08-30

**Authors:** Kimberly F. Macala, Rachel G. Khadaroo, Sareh Panahi, Ferrante S. Gragasin, Stephane L. Bourque

**Affiliations:** 1 Department of Critical Care Medicine, University of Alberta, Edmonton, Alberta, Canada; 2 Department of Anesthesiology and Pain Medicine, University of Alberta, Edmonton, Alberta, Canada; 3 Department of Surgery, University of Alberta, Edmonton, Alberta, Canada; 4 Department of Pharmacology, University of Alberta, Edmonton, Alberta, Canada; Vanderbilt University Medical Center, UNITED STATES

## Abstract

**Background:**

Medication overdose is a prevalent issue and despite mixed reports of efficacy, the use of intravenous lipid emulsions, notably Intralipid^®^, for the management of toxicity from lipid-soluble drugs is becoming increasingly prevalent. Whether alternative lipid emulsion formulations have similar efficacy for resuscitation compared to Intralipid is not known. Here, we compared the efficacy of Intralipid and ClinOleic^®^ for resuscitation following overdose with the lipid-soluble beta-adrenergic antagonist propranolol.

**Methods:**

Male Sprague-Dawley rats (age 3–4 months) were anesthetized with isoflurane and instrumented for direct hemodynamic assessments. In Study One, rats (n = 22) were pre-treated with Intralipid 20% (n = 12) or ClinOleic 20% (n = 10) to determine whether the hemodynamic effects of propranolol could be prevented. In Study Two, rats were randomly assigned to Intralipid 20% (1, 2, or 3 mL/kg IV, n = 21) or ClinOleic 20% (1, 2, or 3 mL/kg IV, n = 20) resuscitation groups following propranolol overdose (15 mg/kg IV). In Study Three the effect of Intralipid 20% (1 mL/kg IV, n = 3) and ClinOleic 20% (1 mL/kg IV, n = 3) in the absence of propranolol was investigated. The primary endpoint in all studies was survival time (up to a maximum of 120 minutes), and secondary endpoints were time to achieve 50%, 75%, and 90% of baseline hemodynamic parameters.

**Results:**

In Study One, pre-treatment with Intralipid prior to propranolol administration resulted in prolonged survival compared to pre-treatment with ClinOleic at low doses (1 mL/kg; P = 0.002), but provided no benefit at higher doses (3 mL/kg; P = 0.95). In Study Two, Intralipid conferred a survival advantage over ClinOleic, with 18/21 rats surviving 120 minutes in the Intralipid group and only 4/20 survivors in the ClinOleic group (P<0.0001). Median survival times (with interquartile ranges) for rats treated with Intralipid, and ClinOleic, and saline were 120 (80.5–120) min, 21.5 (3.25–74.5) min, and 1 (0.25–2.5) min respectively (P<0.001). Only 3/21 rats in the Intralipid group survived less than 30 minutes, whereas 12/20 ClinOleic treated rats had survival times of less than 30 minutes. The number of rats achieving 75%, and 90% of baseline mean arterial pressure was also greater in the Intralipid group (P<0.05 for both values). Treatment in Study Three did not alter survival times.

**Conclusions:**

Low-dose Intralipid (1, 2, or 3 mL/kg IV) confers a survival advantage up to 120 minutes post-propranolol overdose (the end-point of the experiment) and better hemodynamic recovery compared to ClinOleic (1, 2, or 3 mL/kg IV) in rats with propranolol overdose. As health care centres choose alternate intravenous lipid emulsions, limited availability of Intralipid could impact efficacy and success of overdose treatment for lipid-soluble drugs.

## Introduction

Medication overdose is a common and potentially fatal scenario encountered in many specialties.[1]Depending on the pharmacologic agent, management can be complex, lengthy, and costly. Overdose may require intensive care unit (ICU) admission for treatment of serious complications (e.g. arrhythmias, cardiovascular collapse, acute renal failure, and acute liver failure). In the case of severe calcium channel and beta-blocker poisoning, there is no specific antidote, and conventional therapies such as calcium, atropine, or glucagon often fail to improve hemodynamic function,[2] while other commonly instituted treatments (e.g. vasopressors, hyperinsulinemia-euglycemia therapy) can be associated with adverse outcomes. Among experimental agents, intravenous lipid emulsions (ILE) have been successful in resuscitating patients presenting with lipid soluble drug overdoses.[3] ILEs have been a component of parenteral nutrition for several decades.[4] More recently, there has been controversy regarding the off-label use of ILE for treatment of local anesthetic toxicity. Human case reports and animal studies have shown that ILEs may be useful for cardiovascular resuscitation following local anesthetic systemic toxicity,[5,6] although the efficacy of such interventions, particularly compared to conventional resuscitative measures such as vasopressor support,[6] has been questioned.[7] There are also reports surrounding the use of ILE for reversing lipid soluble medication toxicity beyond local anesthetics (e.g. tricyclic antidepressants,[8] calcium channel blockers,[9] beta blockers[10]). Recommendations published recently by the American College of Medical Toxicology[3] and the American Heart Association[11] state that consideration of ILE use at the discretion of the responsible physician is reasonable and may be considered when the standards of care are failing. In a 2016 publication of guidelines for use of ILE for resuscitative purposes in Clinical Toxicology[12], ILEs received neutral recommendations for lipid-soluble beta-blockers in situations of both cardiac arrest and life-threatening toxicity, highlighting the need for more preclinical studies evaluating the safety and efficacy of ILE use as a resuscitative agent.

The most commonly reported ILE used for resuscitation is Intralipid[13] owing to its presence in many medical centres, as well as its promulgation since early studies by Weinberg et al.[14] However, alternative ILE preparations are replacing Intralipid in many health centres, in part based on studies demonstrating that ILEs such as ClinOleic are less pro-inflammatory and lead to better patient outcomes when used for long-term nutritional replacement.[15] Notwithstanding such benefits, it is presently unclear whether such replacement alternative ILEs are as efficacious as Intralipid for resuscitation of lipid-soluble drug overdose. We previously showed that Intralipid 20% (1mL/kg IV) administration is an effective strategy in reversing toxicity with the beta-adrenergic antagonist propranolol in rats.[16] While differences between ILEs in their ability to bind[17,18] and reverse the toxic effects of local anesthetics have been described,[19,20] no such studies have been conducted in the context of beta-blockers. Guidelines currently recommend the use of Intralipid 20% for local anesthetic toxicity, but recommendations for ILE for treatment of non-local anesthetic drug toxicity are neutral.[12]

Here, we sought to determine whether Intralipid or ClinOleic would be associated with improved survival and faster hemodynamic recovery after propranolol overdose in rats. Rats were chosen as an experimental model because of their well-characterized hemodynamic responses to beta blockers.[21] Rats possess beta-receptors with similar affinity to catecholamines as humans,[22,23] and when antagonized with beta blockers, develop marked hypotension (secondary to vasodilation) and bradycardia. This provides a pharmacological endpoint with a clear onset with which we can test reversal of drug toxicity using ILEs. As high doses of ILE can be associated with adverse clinical outcomes (including acute kidney injury, cardiac arrest, ventilation perfusion mismatch, acute lung injury, venous thromboembolism, hypersensitivity, fat overload syndrome, pancreatitis, extracorporeal circulation machine circuit obstruction, allergic reaction, and increased susceptibility to infections,[24,25]) we used doses (1, 2, or 3 mL/kg) of ILE which though lower than those used to treat local anesthetic toxicity in rats (a range of 4-16mL/kg[26,27]) we have shown effectively reverses propranolol toxicity.[16] The primary endpoint was survival time, and secondary endpoints were time to recovery of 50%, 75%, and 90% of baseline hemodynamic parameters.

## Methods

This study was conducted in accordance to the guidelines established by the Canadian Council on Animal Care, with the approval of the Animal Care and Use Committee at the University of Alberta and reported in adherence with the ARRIVE Guidelines. Inhalational isoflurane anesthesia (in 100% oxygen) was used in all experiments described in this study.

### Animals and preparation

Experiments were completed in the same laboratory operating table setting during daytime hours. A total of 73 male procedure- and treatment-naïve Sprague Dawley rats (Charles-River Laboratories International Inc., St-Constant, QC) aged 3–4 months were used in this study. Rats were double-housed in standard shoebox cages containing aspen-chip bedding, nesting material, and PVC tubing for environmental enrichment; cages were kept in the animal care facility at the University of Alberta for a maximum of 4 weeks, which maintained a temperature of 22±1 °C, and a 12h:12h light:dark cycle. Welfare assessments were performed routinely twice weekly by trained animal care staff; no rats developed morbidities or died prior to experimentation. Rats had *ad libitum* access to a standard grain-based rodent chow (PicoLab 5LOD, LabDiet, St. Louis, MO) and tap water.

On the day of the experiment, rats were anesthetized with inhaled isoflurane (induction: 5%, maintenance: 1.5% in 100% O_2_) and kept spontaneously breathing via a nose-cone; rats remained in the surgical plane of anesthesia for the entirety of the experiment, and no rats were allowed to recover. Isoflurane was chosen because it can be administered non-invasively, and depth of anesthesia can be maintained relatively constant with ease. Under anesthesia, rats were instrumented with indwelling catheters in the left femoral artery (PE50; 0.58 mm i.d., 0.97 mm o.d.) and vein (Silastic , Cole-Parmer, Montreal, QC. 0.51 mm i.d., 0.94 mm o.d.) for hemodynamic assessments and drug delivery, respectively. The femoral arterial catheters contained heparinized saline (25 units/mL). Body temperature was monitored via a rectal thermometer and maintained within 36–37 °C with a warming pad. After vessel cannulation, rats were given 30–50 minutes to achieve stable baseline hemodynamics. During this stabilization period, rats received maintenance hydration with sterile normal saline (~2mL·kg^-1^·h^-1^ IV). Oxygen saturation was monitored via pulse oximetry. Arterial pressures and heart rate were continuously monitored and recorded via indwelling catheters connected to a data acquisition system (Lab Chart Pro 8, ADInstruments, Colorado Springs, CO).

### Study One

The primary objective of Study One was to determine whether the provision of ILE prior to administration of propranolol would be able to prevent or diminish the hemodynamic effects of propranolol toxicity and secondly to determine whether Intralipid 20% would have a comparable effect as ClinOleic 20%. After establishing stable baseline hemodynamics, rats (n = 22 total) were randomized to receive a bolus of either Intralipid 20% (1 mL/kg [n = 6] or 3 mL/kg [n = 6] IV) or ClinOleic 20% (1 mL/kg [n = 5] or 3 mL/kg [n = 5] IV) one minute prior to the administration of propranolol (15 mg/kg IV); ILE was administered over a period of 30 seconds, and propranolol was administered over a period of two minutes. Upon completion of the experimental protocol, rats were euthanized by isoflurane overdose and excision of the heart.

### Study Two

The primary objective of Study Two was to establish survival and hemodynamic recovery profiles for Intralipid 20% and ClinOleic 20% resuscitation to a maximum time of 120 minutes following the development of propranolol toxicity. Subsequently, we aimed to determine whether Intralipid 20% would have similar or superior effect compared to ClinOleic 20%. After instrumentation, rats (n = 45 total) were randomly assigned to one of three groups (i) an Intralipid 20% intervention group (n = 21), (ii) a ClinOleic 20% intervention group (n = 20), or (iii) a control (saline; n = 4) group. Each rat received a bolus dose of propranolol (15 mg/kg IV; administered over two minutes); this dose of propranolol invariably causes hypotension and eventual cardiovascular collapse without intervention.[16] Immediately following propranolol administration, rats were given either Intralipid 20% (1 mL/kg [n = 9], 2 mL/kg [n = 6], or 3 mL/kg [n = 6] IV) or ClinOleic 20% (1 mL/kg [n = 8], 2 mL/kg [n = 6], or 3 mL/kg [n = 6] IV), or saline (1 mL/kg [n = 4]) as a bolus over a 30 second period. The starting dose of ILE (i.e. 1 mL/kg) was based on our previous work demonstrating Intralipid 20% could reverse the hypotension and prevent the cardiovascular collapse associated with this dose of propranolol.[16] Following return to baseline blood pressure, rats were monitored for up to two hours following ILE administration, at which time surviving rats were euthanized by isoflurane overdose and excision of the heart. Rats that lived for 120 minutes post-propranolol administration were considered as survivors.

### Study Three

The objective of Study Three was to determine the impact of ILE administration on hemodynamics in the absence of propranolol administration. Rats (n = 6 total) were randomly assigned to receive either three separate doses of 1 mL/kg of Intralipid 20% (n = 3) or ClinOleic 20% (n = 3), with a two-minute wait period between each dose. Hemodynamics were then measured continuously for two hours.

### Outcomes

The primary outcome was survival time following propranolol administration. Secondary outcomes included recovery rate of mean arterial pressure and heart rate, defined by the time to return to 50%, 75%, and 90% of baseline levels. Endpoints were MAP dropping below 80% of baseline for more than 1 minute, or survival for 120 minutes following propranolol administration; in these circumstances, isoflurane concentration was increased to 5% and rats were euthanized by excision of the heart. The total duration of any experiment did not exceed three hours.

### Reagents

Propranolol (Sigma, St-Louis, MO) was dissolved in sterile saline (0.9% NaCl). All propranolol doses were administered at a volume of 1 mL/kg IV. Intralipid 20% was purchased from Fresenius Kabi (Mississauga, Ontario, Canada). ClinOleic 20% was purchased from Baxter Corporation (Mississauga, Ontario, Canada).

### Statistical analysis

Data are presented as either mean±SEM for normally distributed data, or median±IQR for non-normally distributed data. A single animal was considered as the experimental unit (n). Results were obtained from a minimum of 4 rats per group, which enabled us to detect a 40% change in survival time with an estimated error of 20% of the mean, power = 0.80, and α = 0.05. Validation experiments (experiments performed with saline, or ILEs in the absence of propranolol) were performed in a minimum of 3 animals. Continuous hemodynamic parameters (mean arterial blood pressure, heart rate) are plotted in one-minute intervals, and average hemodynamic values were compared by one-way ANOVA with Tukey post-hoc test. Categorical values were assessed by Fisher Exact Test. GraphPad Prism 7 software (La Jolla, CA) was employed for statistical analysis.

## Results

No animals were excluded due to surgical complications or health concerns. One rat was excluded from all data analysis due to a faulty propranolol injection.

### Study One

**Fig 1:**
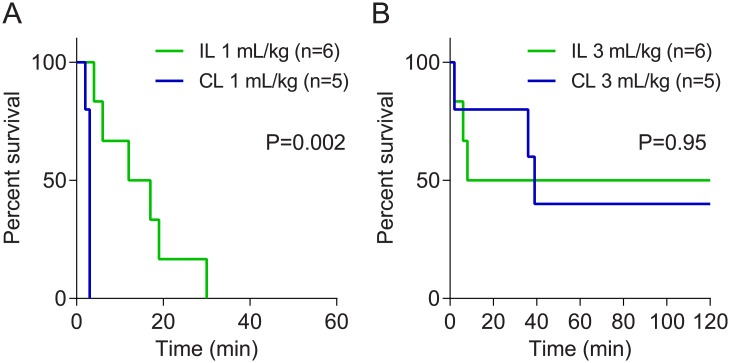
Kaplan-Meier curves depicting survival profiles for rats pre-treated with (A) 1 mL/kg or (B) 3 mL/kg of Intralipid 20% ((IL) or ClinOleic 20% (CL) followed by administration of propranolol (15 mg/kg).

Pre-treatment with 1 mL/kg Intralipid 20% resulted in improved survival times compared to pre-treatment with 1 mL/kg ClinOleic 20% (P = 0.002) (Fig 1A), where average time to death was greater in the Intralipid 20% group (12.6±4.2 min) than with ClinOleic 20% (2.3±0.1 min). Notwithstanding the delayed times until cardiovascular collapse, all rats in both 1 mL/kg pre-treatment groups ultimately succumbed to propranolol toxicity within 30 minutes. Pre-treatment with 3 mL/kg Intralipid 20% resulted in survival of 3/6 rats, and pre-treatment with 3 mL/kg ClinOleic 20% resulted in survival of 3/5 rats (Fig 1B); at this dose, there were no differences in efficacy between either ILE (P = 0.95).

### Study Two

**Fig 2:**
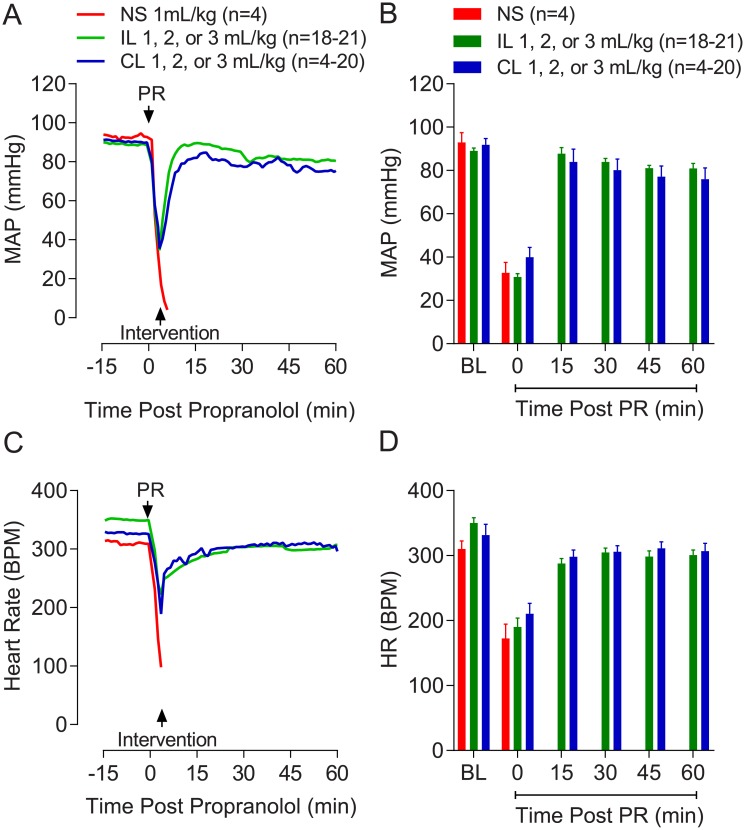
Profiles and summarized values showing (A, B) mean arterial pressure (MAP) and (C, D) heart rate (HR) during 15 minutes of baseline (BL) stabilization following instrumentation, followed by a 60 minute period following propranolol (PR) administration. Only hemodynamic profiles of rats surviving 120 minutes are included for Intralipid 20% (IL) (n = 18/21) and ClinOleic 20% (CL) (n = 4/20) groups; however, all normal saline (NS)-treated rats are shown (none of which survived more than 10 minutes) for reference.

Summarized mean arterial pressure (MAP) and heart rate (HR) traces throughout the experiment are shown in Fig 2. Baseline hemodynamic values were not different between intervention groups (Table 1). Propranolol caused a marked drop in MAP (Fig 2A and 2B) and HR (Fig 2C and 2D).

**Table 1:** Time to recovery to 50%, 75%, and 90% of baseline mean arterial pressure values in resuscitated survivors given 1, 2, or 3 mL/kg intravenous lipid emulsion.

Time (min) to achieve:	Intralipid 20% (1, 2, or 3 mL/kg)	ClinOleic 20% (1, 2, or 3 mL/kg)	P Value
**50% Baseline MAP**	1.8 ± 0.4 (n = 18)	4.0 ± 0.9 (n = 13)	0.03
**75% Baseline MAP**	4.3 ± 1.0 (n = 18)	8.0 ± 1.4 (n = 10)	0.04
**90% Baseline MAP**	5.3 ± 1.0 (n = 17)	14.1 ± 4.9 (n = 7)	0.02

Values are mean±SEM. MAP, mean arterial pressure.

**Fig 3:**
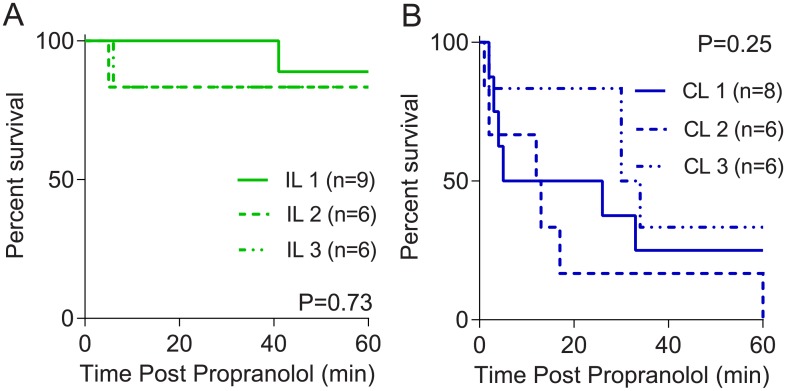
Kaplan-Meier curves depicting survival profiles following propranolol (15 mg/kg) overdose and subsequent treatment with (A) low-dose (1, 2, or 3 mL/kg) Intralipid 20%(IL) and (B) low-dose (1, 2, or 3 mL/kg) ClinOleic 20% (CL).

**Fig 4:**
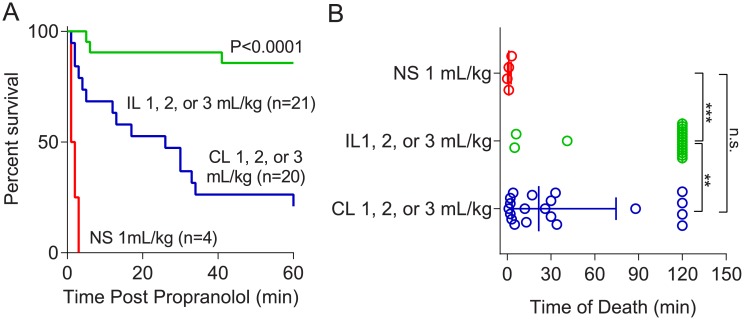
(A) Combined Kaplan-Meier curves depicting survival profiles following propranolol (15 mg/kg) overdose and subsequent treatment with low-dose (1, 2, or 3 mL/kg) Intralipid 20% (IL), low-dose (1, 2, or 3 mL/kg) ClinOleic 20% (CL) and 1 mL/kg normal saline (NS). (B) Median survival times of rats depicted in (A). **P<0.01, ***P<0.0001, n.s. not significant.

Initial experiments were performed to determine whether increasing doses of ILE, starting at 1 mL/kg based on previous experiments,[16] would confer increasing protection. Log-rank tests revealed post-propranolol treatment with 2 or 3 mL/kg ILE provided no survival benefit over 1 mL/kg in either the Intralipid 20% (P = 0.73, Fig 3A) or ClinOleic 20% (P = 0.25, Fig 3B) treated rats. Based on these findings, data for all three doses were pooled, and log-rank-tests were performed to compare Intralipid 20% and ClinOleic 20% treatments (Fig 4).

Intralipid 20% conferred a marked survival advantage compared to saline and ClinOleic 20%; 0 of 4 rats survived in the saline group, whereas 18 of 21 rats survived 120 minutes in the Intralipid 20% group, and 4 of 20 survived in the ClinOleic 20% group. Comparisons between times to cardiovascular collapse following propranolol administration are shown in Fig 4B. Recovery profiles and summarized hemodynamic data of rats that survived 120 minutes after propranolol administration are shown in Fig 2.

Recovery time to 50%, 75%, and 90% of baseline mean arterial pressure was improved in surviving Intralipid-treated rats (Table 1). In the Intralipid 20% group, 18/21, 18/21, 17/21 animals achieved the 50%, 75%, and 90% recovery of baseline mean arterial pressure, respectively, compared to 13/20 (P = 0.16), 10/20 (P = 0.02), and 7/20 (P = 0.004) rats in the ClinOleic 20% group, respectively.

### Study Three

To ascertain whether the hemodynamic effects of ILE treatment were dependent on propranolol toxicity, or instead reflect a non-specific hypertensive effect, rats were treated with ILE in the absence of propranolol administration. Neither Intralipid 20% nor ClinOleic 20% caused any changes in MAP (Intralipid 20%: 0.1±0.3 mmHg, n = 3; ClinOleic 20%: 4.6±0.9 mmHg, n = 3) or HR (Intralipid 20%: 0.7±0.6 beats per minute, n = 3; ClinOleic 20%: 0.9±0.4 beats per minute, n = 3) five minutes post-injection when given in the absence of propranolol.

## Discussion

Intralipid has until recently been a very common lipid in the clinical setting for parenteral nutrition replacement.[28] However, its use has decreased owing to the availability of less inflammatory ILEs for chronic parenteral nutrition.[15] The question remains as to whether alternative ILEs provide similar efficacy for reversal of drug toxicity. Here, rats were administered a non-selective beta-adrenoceptor blocker, propranolol, at a dose (15 mg/kg) previously been shown to be lethal even in rats treated with epinephrine or given fluid resuscitation.[16] While beta-blocker toxicity is a clinically relevant phenomenon (>26,000 cases were reported in the US in 2016, making it one of the top 25 drug overdose categories associated with fatality[29]), severe toxicity due to intravenous administration is a relatively rare event.[29] Notwithstanding, use of an intravenous propranolol model represents an ideal one to study acute lipid soluble medication overdose by virtue of our capacity to monitor a clear onset of cardiovascular collapse and subsequent reversal by intervention in real time. However, there are distinct pharmacokinetic differences between oral and intravenous propranolol administration that warrant consideration given that the vast majority of beta-blocker poisonings are oral. For instance, oral intake is associated with marked first-pass hepatic metabolism resulting in variable bioavailability (up to a 20-fold difference) up to two hours following oral administration, which can influence time of onset of cardiovascular collapse.[30] Furthermore, propranolol clearance is achieved via hepatic metabolism, which results in the formation of 4-hydroxypropranolol, an active metabolite implicated in the enhanced effects when given orally versus intravenously;[30] the effects of different ILEs on 4-hydroypropranolol are unknown, and therefore warrant further consideration.

Both Intralipid 20% and ClinOleic 20%, even at a dose of 1mL/kg, conferred a survival advantage compared to saline administration at a dose of 1mL/kg, confirming our previous results that this effect is attributed to more than fluid administration.[16] We found that Intralipid 20% conferred a survival advantage compared to ClinOleic 20% in terms of survival time, as well as time to recovery of hemodynamics. Clinically, if the benefits of Intralipid 20% observed in this preclinical animal model translates to human overdose patients, it may increase survival by improving hemodynamic stability. Moreover, even in patients wherein Intralipid 20% does not completely restore hemodynamic stability, the benefit of extending survival time may allow time for transfer to the intensive care unit for institution of other potentially life-saving treatments. Despite rats and humans being different species and that intravenous administration of propranolol (as a model of lipid soluble drug toxicity) is uncommon, our results demonstrate an advantage to resuscitation using Intralipid versus ClinOleic, and these results if similar in humans would be of importance in improving survival. Alternatively, with more stable hemodynamics, patients could avoid admission to the ICU altogether, thereby requiring only monitoring (in an observed setting such as the emergency department or in an observation unit) and thus translating into significant cost-savings. These findings support the notion that ILEs can be a life-saving treatment for patients experiencing cardiovascular collapse associated with a lipid soluble drug overdose,[4] but the composition of the ILE is an important factor to consider. Thus, health practitioners may be remiss in replacing Intralipid 20% with other ILEs based solely on the nutritional benefits, without considering the resuscitative properties of various formulations.

The finding that no dose-related trend was observed with ILE administration is consistent with our previous pilot studies indicating doses higher than 1 mL/kg for Intralipid 20% do not confer additional benefits in the wake of propranolol toxicity.[16] The doses used herein are lower than those typically reported for treating local anesthetic toxicity in rats (typically between 6–16 mL/kg)[31]. We reasoned that if low doses of ILE could effectively reduce toxicity, higher doses may be associated with side-effects that offset the survival advantage. From a clinical standpoint, lower doses (and hence smaller administered volumes) are more likely to be tolerated by patient populations with compromised cardiovascular function (e.g. congestive heart failure) and may explain why higher doses did not confer additional protection.

The common purported lipid sink hypothesis posits that an expanded lipid phase acts to partition a lipid soluble drug away from its site of action, thereby lessening its toxic effects and increasing its metabolism.[32] Alternative mechanisms of action of ILEs have also been proposed, which include direct effects on the myocardium, relating to inotropic effects and improving cardiac cell survival.[33–35] However, we previously showed that low-dose Intralipid 20% (1 mL/kg) does not improve cardiac contractility or blood flow, either when administered alone or immediately following propranolol toxicity.[16] These data may suggest at low doses, ILEs act predominantly to alter the pharmacokinetic profile of lipid soluble drugs, whereas at high doses the direct cardiac effects manifest; this idea is consistent with the observation that high doses of Intralipid (16 mL/kg), despite improving cardiac sodium channel blockade (measured by QRS prolongation), did not improve overall survival in the wake of toxicity with the water-soluble beta-blocker atenolol.[36]

As a lipid sink, the composition of these ILEs, particularly the composition of fatty acids, is likely to have important implications.[15] ClinOleic 20% (containing olive oil and soybean oil), consists predominantly of mono-unsaturated fatty acids. Intralipid 20% contains only one oil (soybean oil) with predominantly long-chain poly-unsaturated fatty acids. The composition of Intralipid 20% may provide better sequestration of lipid-soluble agents than other ILEs consisting of a greater proportion of medium chain fatty acids.[18,20] However, Evans *et al*. showed no such differences between Intralipid and ClinOleic,[37] and in fact a recent study by Ruan et al. showed that the ILE Lipofundin, which consists of a mixture of medium and long- chain fatty acids binds to lipid soluble local anesthetics better than Intralipid.[17] Thus, factors beyond the lipid composition of ILEs need to be considered to reconcile these apparent disparities. In the present study, a threefold higher dose of ClinOleic 20% (3 mL/kg) conferred no additional benefit following propranolol overdose (Fig 2A), suggesting the difference between Intralipid 20% and ClinOleic 20%, at least at low doses, is not related to potency.

In study one, pre-treatment with ILEs was conducted to assess whether ILEs could prevent or mitigate the hypotension and subsequent cardiovascular collapse associated with propranolol toxicity. Although this information is of limited clinical value due to the inability to predict beta-blocker toxicity and the rapid time course in which cardiovascular collapse can occur, these experiments provide some additional insights into the mechanism of action. If an expanded lipid phase is all that is necessary for reversal of toxic effects, then provision of pre-treatment ILE should at least alleviate the toxic symptoms. While pre-treatment with Intralipid 20% delayed mortality compared to ClinOleic 20% at 1 mL/kg, all rats ultimately succumbed to the propranolol toxicity by 30 minutes. Interestingly, with pre-treatment with doses of 3 mL/kg, both Intralipid 20% and ClinOleic^®^ 20% improved survival, and no between group survival advantage was found. We cannot provide a definitive explanation for why the higher dose of ILE was more efficacious than the lower doses with the pre-treatment protocol, whereas no such differences were observed in the post-treatment phase (study two). However, it is tempting to speculate that, if Intralipid 20% and ClinOleic 20% confer survival advantage by acting as a lipid sink, a greater dose would ensure therapeutic levels of ILE despite the ILE re-distribution into the periphery and extravasation that would occur prior to propranolol administration.

There are several limitations of the study that should be considered. In our study design the timing of ILE administration is immediately before or after intravenous propranolol injection. While intravenous beta blocker toxicity is uncommon, it does provide a useful model of hypotension, but may make our findings less generalizable to human poisonings. In a less acute oral ingestion overdose in a human, the pharmacokinetic profiles are likely more variable, and the involvement of other products of metabolism (e.g. 4-hydroxypropranolol) further confound the findings. A second limitation of this study was the inability to assess cardiac contractility independent of preload and afterload; as such, changes in hemodynamics could reflect combined changes in cardiac output and total peripheral resistance. However, as indicated above, we previously showed that the doses of Intralipid 20% used herein did not impact cardiac contractility or heart rate, even following beta-blocker overdose.[16] However, assessment of blood pressure does not provide information about regional blood flow patterns or the degree of tissue perfusion, and this information could provide insights into the means by which Intralipid 20% confers a survival advantage over ClinOleic 20% in the wake of propranolol toxicity. Third, experimenters were not blinded to the treatment groups, although the use of categorical endpoints and objective measures limit bias. Finally, it is also important to acknowledge that due to the exploratory nature of this study, accurate estimates of means, dispersions, and differences in survival times were not available from the outset, and therefore final group numbers were based on interim data analyses. This resulted in unbalanced group numbers, and future studies using *a priori* power calculations based on the parameters collected here are warranted.

In conclusion, our findings supplement the current knowledge that ILE is a beneficial treatment in propranolol overdose for restoration of hemodynamic stability. We have newly demonstrated that Intralipid 20% treatment provides a survival advantage compared to ClinOleic 20% for resuscitation from propranolol toxicity. These findings suggest that replacement of Intralipid 20% by alternative ILEs in the ICU warrants careful consideration.
